# Moderate alcohol use is associated with decreased brain volume in early middle age in both sexes

**DOI:** 10.1038/s41598-020-70910-5

**Published:** 2020-08-19

**Authors:** Satu Immonen, Jyrki Launes, Ilkka Järvinen, Maarit Virta, Ritva Vanninen, Nella Schiavone, Eliisa Lehto, Annamari Tuulio-Henriksson, Jari Lipsanen, Katarina Michelsson, Laura Hokkanen

**Affiliations:** 1 Department of Psychology and Logopedics, Faculty of Medicine, University of Helsinki, Helsinki, Finland; 2 Institute of Clinical Medicine, Radiology, University of Eastern Finland, Kuopio, Finland; 3 Department of Clinical Radiology, Diagnostic Imaging Centre, Kuopio University Hospital, Kuopio, Finland; 4 Independent researcher, Helsinki, Finland

**Keywords:** Neuroscience, Neurology

## Abstract

The aim was to examine cross-sectional association between moderate alcohol consumption and total brain volume in a cohort of participants in early middle-age, unconfounded by age-related neuronal change. 353 participants aged 39 to 45 years reported on their alcohol consumption using the AUDIT-C measure. Participants with alcohol abuse were excluded. Brain MRI was analyzed using a fully automated method. Brain volumes were adjusted by intracranial volume expressed as adjusted total brain volume (aTBV). AUDIT-C mean of 3.92 (*SD* 2.04) indicated moderate consumption. In a linear regression model, alcohol consumption was associated with smaller aTBV (*B* = − 0.258, *p* < .001). When sex and current smoking status were added to the model, the association remained significant. Stratified by sex, the association was seen in both males (*B* = − 0.258, *p* = 0.003) and females (*B* = − 0.214, *p* = 0.011). Adjusted for current smoking, the association remained in males (*B* = − 0.268, *p* = 0.003), but not in females. When alcohol consumption increased, total brain volume decreased by 0.2% per one AUDIT-C unit already at 39–45 years of age. Moderate alcohol use is associated with neuronal changes in both males and females suggesting health risks that should not be overlooked.

## Introduction

Alcohol abuse is harmful to the brain, but the effects of moderate consumption, and the earliest age when the harmful effects appear, are less known. Volume changes of small parts of the brain, e.g. hippocampus^1^ and the ventricles^2,3^, have been associated with moderate consumption, but the relationship of alcohol consumption and total brain volume is contradictory. Methodological differences in imaging technology and in estimating alcohol consumption may make the results difficult to compare and interpret. In addition, most previous studies of global brain values have included participants in late middle-age or old age. However, the rate of age-related brain volume does not occur in a linear fashion, but the decrease accelerates towards old age making the effect of age difficult to control in statistical analysis. Studying younger participants and groups with less age heterogeneity would be helpful.

We found ten studies^4–13^ which used volumetry, voxel-based morphometry or brain age estimates for analyzing the association of large brain structures and alcohol consumption in moderate drinkers (Table 1). Two of these studies included middle-aged participants in their forties to sixties^4,5^, two mainly over 65-year-olds^6,7^, and six report on participants of a wide age range^8–13^. Findings in the middle-aged were conflicting. Decreased^4^ and increased^5^ volume of white matter, and increased volume of gray matter and ventricular size were associated with moderate drinking in males, but no associations were found in females. Studies of participants over 65 years found either larger^6^ or smaller^7^ whole brain volumes associated with drinking. Studies of a wider age range have found decreased volumes^8–10^ or no association^11,12^ with consumption. The most recent study using the UK Biobank data found an association between brain ageing and daily or almost daily consumption of alcohol, but the association was not significant in lower levels of alcohol use^13^.

**Table 1:** Previous MRI studies on moderate drinking and brain structure using volumetry, voxel-based morphometry or brain age estimates. Only studies that examined global brain structures (e.g. total brain, total gray matter, total white matter or total cerebrospinal fluid) are included.

References	Year	Origins of sample	N	% Male	Age	Alcohol consumption	Tissues examined	Main association with alcohol consumption^a^
^4^	2006	PATH Through Life Project	385	55	Range 60–64	Drinks per week	GM, WM, CSF	Males: Larger ventricular volume and GM. Smaller WM Females: Equivocal findings for CSF and WM
^5^	2005	Paid volunteers recruited by newspaper advertisements	91	52	Male M (SD) = 49.5 (8.7) Female M (SD) = 49.9 (6.9) Range not given	Lifetime intake. Drinks per week	GM, WM, CSF, TBV	Males: Larger WM (lifetime intake) Females: No significant effect
^6^	2014	Clinical sample from insurance beneficiaries	589	33	≥ 65 M (SD) = 80.1 (5.5) Range not given	Drinks per month categorized into non-drinkers and light-to-moderate drinkers	TBV	Larger TBV in a sample of both males and females
^7^	2014	AGES- Reykjavik study	3,363	41	M (SD) = 74–77 (SD = 4.7–5.4) depending on group Range not given	Drinking status Drinks per week categorized into 2 or 3 levels from very light to moderate drinkers	TBV	Males: Smaller TBV Females: No significant effect
^8^	2008	Framingham heart study	1839	47	M (SD) = 60.64 (9.42) Range 33–88	Drinks per week categorized into 5 levels from non-drinkers to heavy drinkers	TBV	Smaller TBV in a sample of both males and females Males: Smaller TBV Females: Smaller TBV
^9^	2009	Community volunteers from rural area, Japan	385	39	≥ 40 M (SD) = 67.2 (11.8) Range not given	Drinks per week categorized into 3 levels from non-drinkers to moderate drinkers	TBV	Smaller TBV in a sample of both males and females
^10^	2006	Selected volunteers for a database of normal MRI images	405	100	M (SD) = 46.98 (14.56) Range 18–81	Lifetime intake	GM	Males: Smaller GM
^11^	2009	Volunteers without alcohol dependence Recruitment population not given	211	54	M (SD) = 37.4 (13.5) Range 21–72	Lifetime intake	GM, WM	Males: No significant effect Females: No significant effect
^12^	2014	Normal scans selected from neurological patients	367	41	≥ 18 M (SD) = 53 (13) Range not given, but 69% were older than 45	Drinks per week categorized into 3 groups. Each group was compared to a matched group of abstainers. Also, all drinkers and abstainers were compared	GM, WM	No significant effects in samples of both males and females
^13^	2020	UK Biobank	12,115	47	M (SD) = 63.3 (7.4) Range 45–80	Current alcohol intake frequency categorized into 6 levels	Brain Age calculated from brain morphometrics	Daily or almost daily consumption of alcohol was significantly associated with increased brain ageing in a sample of both males and females

*TBV* total brain volume, *GM* Gray Matter, *WM* White Matter

^a^In studies which examined both local and global volumes, we listed results from global volume analyses only.

The amount to which the brain normally occupies the intracranial cavity varies considerably with age even in healthy subjects. Brain volume decreases with age in adults^14,15^, but is most stable around the age of forty years^14^. As confounding cerebrovascular and neurodegenerative conditions are also infrequent at this age, it is an optimal age to study how alcohol intake associates to brain volume. We studied the association of moderate alcohol consumption and total brain volume in a birth risk cohort aged 39 to 45 years in males and females.

## Methods

### Sample

The participants were part of a consecutive cohort that initially included 1196 infants born with various birth risks in a single hospital in Helsinki between 1971 and 1974^16^. A total of 202 children died or had severe disabilities, e.g. cerebral palsy, blindness or Down syndrome, and were excluded from the cohort, leaving 994 participants for prospective follow-up^16,17^. The inclusion criteria were hyperbilirubinemia (bilirubin level > 340 µmol/l or blood transfusion), birth weight below 2000 g, Apgar score < 7, respiratory distress requiring external ventilation, maternal diabetes, hypoglycemia (blood glucose ≤ 1.67 mmol/l), septicemia, or severe neurological symptoms such as rigidity, apnea, hyperexitability, convulsions or prolonged feeding difficulty in the absence of other risks^16,17^. In addition to the birth risk cohort, 164 singletons without birth risks have been followed from childhood as controls. The previous follow-up investigations were conducted at 5, 9, 16, and 30 years of age both among the birth risk cohort and control participants^17^.

During 2014–2016, a total of 414 members of the birth risk cohort and 83 controls agreed to be re-examined. They were 39–45 years old community-dwelling adults with normal school history. The follow-up assessment consisted of a neurological and a neuropsychological examination, MRI, and a 516-item questionnaire (available from^18^) which also included the Alcohol Use Disorders Identification Test (AUDIT)^19^.

A total of 393 participants had both brain MRI at a single imaging facility in Helsinki and a completed AUDIT test. We excluded 40 participants for following reasons: alcohol abuse (n = 5), poor quality MRI (n = 3), volBrain image analysis not available (n = 2), neurological conditions or MRI abnormalities e.g. multiple sclerosis, traumatic brain injury, ventriculomegaly, or severe white matter changes (n = 21), severe psychiatric problems (n = 9). See below for sources of information. The final sample consisted of 353 participants (163 males and 190 females).

Of the 353 participants, 289 had a history of birth risks, including hyperbilirubinemia (n = 84), a low Apgar score or respiratory distress (n = 79), birth weight < 2000 g (n = 67), hypoglycemia (n = 18), maternal diabetes (n = 20), and neurological symptoms (n = 21). The remaining 64 participants were controls not exposed to birth risks.

### Demographic and health data

Health and demographic data were acquired with the 516-item questionnaire filled out either online or as a mailed survey and the medical examination. Hospital and outpatient clinic discharge diagnoses (ICD-8, ICD-9 and ICD-10; 1975 onwards) were collected from the Care Register for Health Care (HILMO) of the Finnish Institute for Health and Welfare^20^. Purchases of reimbursed prescription drugs were obtained from the Social Insurance Institution of Finland^21^. A total of 92% of participants consented to share their registry information. Cerebrovascular events and risks, i.e. hypertension, diabetes, hyperlipidemia, high Body Mass Index, and heart disease, were identified by a neurologist (J.L.) using given history, a clinical examination, questionnaire, and registry data. Other neurological conditions, alcohol abuse and psychiatric problems were evaluated by J.L. using all the above information. Wechsler Adult Intelligence Scale–Fourth Edition^22^ was used to measure the full-scale IQ. Education was classified into three levels: basic education, including the obligatory 9 years, secondary, including completed high school, vocational or comparable education, which lasts typically 12 years, and higher education, e.g. university-education.

### Alcohol consumption

The questionnaire included a Finnish translation of the AUDIT^19^. AUDIT-C, a three-item short version derived from AUDIT, was used as the measure of alcohol consumption^23^. AUDIT-C questions assess how often and how much one typically drinks and how frequently binge drinking occurs (range 0–12). The second AUDIT-C question “How many drinks containing alcohol do you have on a typical day when you are drinking?” includes response options ranging from 1 to 10 or more drinks. Response option 0 drinks (scored as 0) was added to capture responses from nondrinkers. AUDIT-C scores of 3 or below in females and 4 or below in males have been used to indicate low-level drinking, scores of 5–8 to indicate possibly harmful but moderate alcohol use, and scores of 9 or higher to indicate potential alcohol abuse^24,25^. Five single-item responses were missing in AUDIT-C and were imputed with zero.

### Other substance use

Current smoking, lifetime cannabis and illicit drug use were assessed on the questionnaire in yes/no format. Smoking status was classified as a current smoker or current non-smoker. Eight participants did not provide information on cannabis use and nine participants on lifetime illicit drug use. Thirty nine participants did not provide information on current smoking, 22 of whom had reported to be non-smokers in a questionnaire given ten years earlier. All were included as non-smokers.

### MRI acquisition and volumetric analysis

Brain MRI scans were obtained using two 1.5 T MRI scanners (Signa; General Electric, Milwaukee, USA). Sequences included a T1-weighted three-dimensional structural sequence (Cube), used for volumetric analysis. Parameters used were: time of repetition (TR) 540 ms, time to echo (TE) 9.9 ms, flip angle 90 degrees, spacing between slices 0.59 mm, pixel spacing 0.48/0.48 mm, slice thickness 1.2 mm, and acquisition matrix 256 × 256. Additionally, T2-weighted fluid attenuation inversion recovery (FLAIR), susceptibility weighted imaging (SWAN), and either diffusion weighted imaging or 30-direction axial diffusion tensor imaging (half of all cases) sequences were performed. All scans were visually assessed by an experienced neuroradiologist (R.V.) who was blinded to clinical data and volumetric quantification. Imaging results with all sequences evaluated were, with regard to age, normal in 332 (94%) while 21 participants had clinically irrelevant minor abnormalities (e.g. developmental venous malformations, microbleeds, non-specific hyperintense white matter changes, or small cerebellar infarction).

Brain volume was analyzed using the fully automated MRI volumetry system volBrain^26^, which analyzes 3D T1-weighted scans to calculate volumes of total brain tissue, gray matter, white matter, cerebellum, hippocampus, lateral ventricles, thalamus, caudate nucleus, putamen, globus pallidus, nucleus accumbens, and amygdala. We used the total brain volume with cerebellum and brain stem included. To control for individual differences in overall cranial size, adjusted total brain volume (aTBV) was calculated by dividing brain volume by intracranial volume and expressed as a percentage.

### Statistical methods

Based on power calculations the required sample size to detect a regression coefficient 0.2 with 80% power was 184 and with 95% power 304. Power calculations were based on simple regression analysis between alcohol consumption and aTBV assuming 5% alpha level and using observed standard deviations AUDIT-C (SD = 2.42) and aTBV (SD = 2.38) which were calculated from all available cases. Power calculations were done using the G*power 3.1.9.2^27^.

Linear regression was used to examine the association between alcohol consumption and aTBV, first unadjusted and then adjusting for covariates. Results are given as unstandardized coefficients (*B*) with 95% CI. Age, sex, birth risk status, current smoking, and the neuroradiological status (normal/abnormal) were potential confounders. Those found to be associated with aTBV using ANOVA, *t*-tests or Pearson’s correlation were included as covariates in the adjusted model. Normal distribution of residuals and equality of variances were confirmed via visual inspection of residual plots and histograms. All tests were two-tailed. Statistical analyses were calculated using R version 3.4.4^28^.

### Standard protocol approvals, registrations, and patient consents

Ethical approval was obtained from the Ethical Review Board of the Helsinki and Uusimaa hospital district (Number 147/13/3/00/2013). The participants gave written informed consent. All methods were carried out in accordance with relevant guidelines and regulations.

## Results

Characteristics of the sample are given in Table 2. AUDIT-C mean indicated low-level to moderate use. Ninety participants had cerebrovascular risks, but none had cerebrovascular events. Males consumed more alcohol than females, *M* = 4.88 (*SD* = 1.92) vs. *M* = 3.10 (*SD* = 1.77) respectively, *t*(351) = 9.073, *p* < 0.001. Smokers consumed more than non-smokers, *M* = 4.94 (*SD* = 1.99) vs. *M* = 3.69 (*SD* = 1.98) respectively, *Welch t*(99.014) = − 4.646, *p* < 0.001.

**Table 2:** Sample characteristics (n = 353).

	Mean	SD	Range	n
Age	42.02	1.34	39–45	353
BMI	26.29	5.03	16.76–45.17	351
IQ	107.44	17.51	40–141	349
AUDIT-C	3.92	2.04	0–10	353
aTBV	85.62	2.13	79.08–90.43	353

	n	%		
**Sex**				
Males	163	46		353
Females	190	54		
**Education**				
Basic education	26	7		353
Secondary education	195	55		
Higher education	132	37		
Not drinking alcohol	13	4		353
Cannabis use^a^	29	8		345
Other illicit drug use^a^	10	3		344
Currently smoking	67	20		336
Cerebrovascular risks	90	25		353
Hypertension	65	18		
Diabetes	10	3		
Hyperlipidemia	18	5		
Other heart condition	16	5		

*BMI* Body Mass Index, *AUDIT-C* Alcohol Use Disorders Identification Test-Consumption, *aTBV* adjusted Total Brain Volume.

^a^Lifetime use.

Age did not correlate with aTBV,* r* = -0.013, *p* = 0.805, and aTBV did not differ between participants with scans with no (n = 332) and with minor abnormalities (n = 21),* M* = 85.66 (*SD* = 2.14) vs. *M* = 84.98 (*SD* = 2.00) respectively, *Welch t*(22.978) = 1.486*, p* = 0.151. Therefore, age or MRI status were not included as a covariate. Also, there were no differences in aTBV between the birth risk groups (including the control group) according to the ANOVA [*F*(6,346) = 1.988, *p* = 0.067] and Tukey’s pairwise comparison test. Females had a larger aTBV than males with a mean difference of 0.63 percentage point, *t*(351) = 2.780,* p* = 0.006, and smokers had smaller aTBV compared to non-smokers with a mean difference of 0.63 percentage point, *Welch t*(93.862) = 2.088, *p* = 0.040. Therefore, sex and smoking were used as covariates.

**Figure 1:**
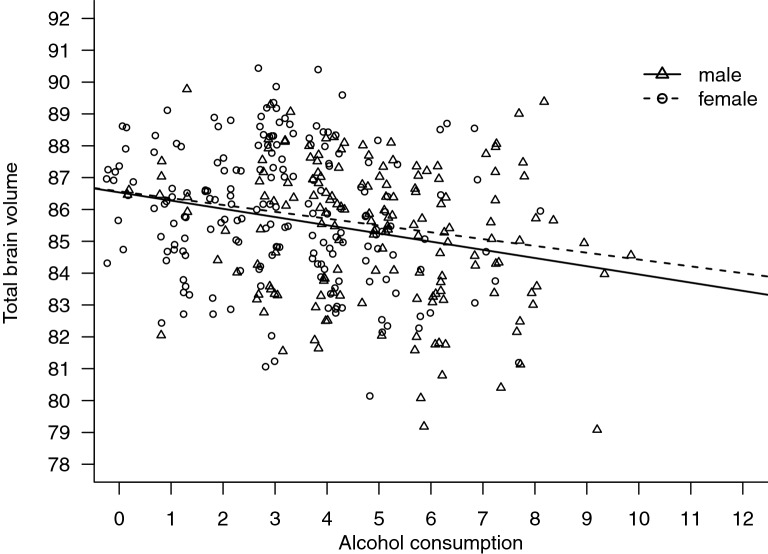
Association between AUDIT-C and aTBV. Association between alcohol consumption (AUDIT-C as a continuous variable) and total brain volume adjusted for intracranial volume in males and females. Points in the plot have been jittered to improve visibility of single cases.

The unadjusted association of alcohol consumption and aTBV was significant with *B* = − 0.258 (Table 3). When sex and current smoking status were added to the model, the association of alcohol consumption with the aTBV remained statistically significant (see Table 3). When the analysis was stratified by sex (Fig. 1), the association was seen in both males *B* = − 0.258 (− 0.429, − 0.086), *p* = 0.003 and females *B* = − 0.214 (− 0.379, − 0.049), *p* = 0.011. Adjusted for current smoking, the association was observed in males *B* = − 0.268 (− 0.444, − 0.091), *p* = 0.003, but not in females *B* = − 0.159 (− 0.330, 0.011), *p* = 0.066.

**Table 3:** Associations between alcohol consumption (AUDIT-C) and total brain volume adjusted for intracranial volume (aTBV).

	*B*	95% CI		*p*
**Unadjusted regression model**				
AUDIT-C	− .258	− .364	− .152	< .001
**Adjusted regression model**				
AUDIT-C	− .216	− .338	− .094	.001
Sex (Male)	− .238	− .723	.248	.336
Current smoking	− .358	− .930	.215	.220

*B* unstandardized regression coefficient beta.

## Discussion

A higher amount of alcohol consumption was associated with a smaller brain volume among moderate drinkers aged 39 to 45 years. The effect persisted after adjusting for sex and smoking, and it appeared in males and females independently. A one-unit increase in AUDIT-C corresponded to approximately a 0.2% reduction in total brain volume. Previously, the association of moderate alcohol consumption and brain volume in early middle-age has been unclear especially in females.

Two previous studies found smaller total brain volume associated with moderate drinking in a combined group of males and females with a mean age of over 60 years^8,9^. In these studies, the magnitude of the total brain volume difference between non-drinkers and moderate drinkers was approximately 1%. These are the only findings in line with our results. Two other studies of total brain volumes have reported inconsistent findings^5,6^. The first study of moderately drinking adults mainly around their forties and fifties found no reduction in total brain volume^5^, but the sample size (n = 91) was considerably smaller than in our study. The second study found a larger total brain volume associated with moderate drinking^6^, but the participants were around 80 years of age. Small effects may be difficult to differentiate from age-related variability when the sample is small or includes elderly people. For comparison, normal age-related total brain volume decline is approximately 0.4% per year after the age of 60 years^14,15^, but very slow around 40 years, i.e., in the age of our group. Brain ageing has recently been found to accelerate with daily or almost daily consumption of alcohol in a sample of participants above the age of 45^13^.

Interpretation of most previous studies is challenging because of small sample sizes, selection bias due to questionable recruitment methods (e.g. newspaper advertisements), and incomplete data of concomitant conditions. Also, many of the studies use complex regression models which include a large number of covariates in the analysis, which causes concerns of over-fitting and insufficient statistical power^29^. We confirmed by power calculations that we had a sufficient number of participants for analyzing aTBV at a satisfactory statistical power. The effect of alcohol use on smaller brain structures (e.g. hippocampus) has been analyzed in several studies^1^. We calculated that using the same power assumptions in our volBrain analysis, including, for instance, the hippocampus would have required 1009 participants (slope = 0.0017, *SD* = 0.046). The increasing use of open datasets and biobanks will help in achieving adequate sample sizes.

Methods of estimating the amount of consumed alcohol vary between studies. Commonly used methods include the number of drinks consumed per week^4,5^, often categorized into drinking groups^6–9,12^, and lifetime intake in kg^5,10,11^ (Table 1). The AUDIT-C score measures the frequency and quantity of drinking as well as the frequency of heavy drinking occasions^23^. Although they are not directly comparable, all methods give estimates of consumption which can be used in regression analysis. The current sample had a group mean indicating low-level use, comparable to the levels found in a Finnish population-based study^30^. Many of the alcohol related health risks are linear^31^. All cutpoints are therefore arbitrary but still often needed in clinical use. In the Finnish study, the highest specificity for heavy use (defined by 16 drinks per week in males and 10 in females) was reached with an AUDIT-C cutpoint of 9 in males and 7 in females, while cutpoints of 7 and 5 were suggested for better sensitivity^30^. Others have also found scores of 9 or higher to indicate potential alcohol abuse and related health risks^24,25^. In our sample only four individuals reported a high score. Those with confirmed abuse were excluded.

Males consumed more alcohol and they also had larger brain volumes, and therefore the effect of sex was analyzed in more detail. Based on the results, the rate of change was of the same magnitude among sexes. In earlier studies, association between gray matter volume and alcohol use has been demonstrated in males^10^. However, females with alcohol use disorder appear equally susceptible to alcohol related brain damage as males, e.g. in the Framingham cohort study, that included a wide range of ages and consumption levels, the association between alcohol consumption and brain volume was actually more pronounced in women than in men^8^. The sex difference in susceptibility at low consumption levels or at a younger age has not been demonstrated. In the age range of our study, the existing studies have not shown unequivocal association between alcohol use and global brain volume in women^5,10,11^. In our sample, the association was visible in both sexes, analyzed combined as well as separately. When stratified by sex, a difference was seen only in the effect of smoking status. Current smoking is associated with a decreased brain volume^32^ and increased brain ageing^13^. Current smokers also in our study had a smaller brain volume, as compared to non-smokers. However, smokers also consumed more alcohol, which probably, at least in part, explains this result. Smoking status did not explain the association between brain volume and alcohol consumption when both sexes were included.

A key strength of our study is that our participants come from a socio-economically and medically very homogenous longitudinal cohort, and they have been followed from birth. Participants were nearly the same age, resulting in minimal age-related variability which enhances the sensitivity to detect small effects. We also had previous and current medical registry data, self-reports, and a clinical examination to identify concomitant factors and exclude major conditions. None of the included participants had marked abnormalities in the four MRI sequences that were used. The imaging quality was ensured by using scans from a single facility and excluding scans with suboptimal quality. A fully automatic volumetric segmentation, unbiased by subjective user input, was used^26^. Further, we refrained from analysis of the small brain structures, as sample size calculus indicated insufficient statistical power.

Limitations may be caused by the cohort’s structure, the measurement of alcohol consumption, and the choice of volumetric technique. Eighty-two percent of participants had a history of a birth risk. Thus, it is not a customary population sample cohort. However, participants with severe consequences or disabilities were excluded already in childhood and aTBV did not differ between different birth risks or controls. The participants were living independently, mostly with jobs and, according to Statistic Finland^33^, an education level corresponding to the general population of Finland. We think that it is very unlikely that birth risk history would affect our results. The estimates of alcohol consumption were based on self-reported typical consumption using AUDIT^19^, which may be prone to under-reporting^34^. However, we were able to identify and exclude excessive drinkers using the comprehensive medical data. Using volBrain for volume measurements may complicate comparison with other studies conducted with e.g. the commonly used FreeSurfer. We chose volBrain because, in our study, accurately measuring the ratio of total brain volume to intracranial volume was important. The estimated intracranial volume from FreeSurfer may be biased by total brain volume^35^ and also the Freesurfer manual suggests using another method for determining the intracranial volume^36^. Further, volBrain is light on computing resources and the stability of brain volume in the middle age has been compellingly demonstrated using volBrain^14^.

## Conclusions

We found a direct association between moderate alcohol consumption and decreased brain volume at early middle-age in both males and females. Understanding of the mechanisms of moderate drinking on the brain is incomplete, but even moderate alcohol consumption may have a harmful effect already in middle-age. Recent systematic analysis on alcohol use and global disease burden suggests that the level of consumption that minimizes health loss is zero^31^. The risk that even moderate drinking poses on the brain should not be overlooked.

## Data Availability

Restrictions apply to the data. Although the participant-level data do not include participant identification, the ethics review board decision demands confidentiality. Pseudonymized data are available to a qualified investigator from the corresponding author.
